# The Treatment and Management of Asthma

**Published:** 1893-01

**Authors:** Thomas J. Mays

**Affiliations:** Professor of Diseases of the Chest in the Philadelphia Polyclinic, and Visiting Physician to the Rush Hospital for Consumption, Philadelphia


### THE TREATMENT AND MANAGEMENT OF ASTHMA.*

BY THOMAS J. MAYS, M. D.,
Professor of Diseases of the Chest in the Philadelphia Polyclinic, and Visiting Physician to the Rush Hospital for Consumption, Philadelphia.

Asthma is a paroxysmal disease of the pneumogastric nerves which throws the muscular fibers into spasmodic contraction. Its prominent symptoms are itching of the head and neck, oppression and tightness of the chest, dyspnœa, bloating of the abdomen, pains in the region of the diaphragm, cough, expectoration and fevers. Its causes are predisposing and exciting. (1) It may be inherited as asthma, and it may appear in children who come from consumptive or nervous families. It seems as if there is a predisposition necessary before the disease can develop. (2) Among the exciting causes are the inhalation of dust, powdered ipecacuanha, pollen of grasses and of roses, odors of certain animals, as cats, sheep, etc. Reflex excitation coming from the nose, stomach, liver, intestines, uterus, etc. Its relation to hay fever is very close. Practically there is no difference be-

*An abstract of a lecture delivered to the class in the Philadelphia Polyclinic, November, 1892.

tween the two. I find that that which relieves the one will also relieve the other.

Its treatment resolves itself into (1) that which aims to give immediate relief from the paroxysm, and (2) that which aims to prevent a recurrence of the paroxysm.

Those remedies which relieve the paroxysms may be classified as follows: (1) Central narcotics, consisting of morphine, belladonna, stramonium, hyoscyamus, tobacco, chloroform, ether, ethyl bromide, etc.; (2) emetics, consisting of lobelia, ipecacuanha, sanguinaria, etc., and (3) the peripheral narcotics or relaxants, consisting of nitroglycerine, amyl nitrate, sodium nitrate, pilocarpine, etc. Now, all our more or less powerful therapeutic agents are stimulants to the general or special bodily tissues which they affect, in small doses, while in large doses they paralyze the same. All the above named agents only relieve asthma when given in large or paralyzing doses—the central narcotics exerting their influence on the central nervous system, the emetics acting on the pneumogastric filaments, while the peripheral narcotics paralyze the vaso motor or sympathetic nerves which supply the unstriped muscular fibers of the bronchial mucous membrane and blood vessels. While all these agents relieve asthma, and, indeed, in some cases are indispensable, it is quite clear that in doing so they lower or depress the functions of the parts on which they act, and that they do not, therefore, come up to the ideal of an asthma remedy. The best among them are nitroglycerine, one or two minims of a one per cent. solution every three or four hours, by the mouth, and $\frac{1}{20}$ or $\frac{1}{16}$ of a grain of morphine hypodermically once or twice a day.

What then is the remedy which may be given continuously for the alleviation of this disease, and without the undesirable effect of the above named classes? Which drug will relieve asthma in stimulant doses? Such a drug, I believe we possess in strychnine. Of course we must bear in mind that all stimulants are only supplementary agents which maintain the functions of the body without adding any direct material support to the same; but there is also good reason for believing that they cause the tissue to appropriate a larger amount of nutritive material than they would otherwise do; and in this way our stimulant drugs become tissue-builders. It has been shown that the power of strychnine in this respect is greater than that of any other stimulant. This drug has a special affinity for the nervous system, which action is especially accentuated on the respiratory center and pneumogastric nerves. In stimulant doses it gives a supporting influence to the respiratory movements and unlike morphine, lobelia, belladonna or nitroglycerine, it does not depress or narcotize the nervous system. Asthma being a spasmodic disease in what manner does stychnine bring relief ? How does it act as an anti-spasmodic ? The most probable theory of the spasmodic state is that there is at the beginning of the program a superabundant discharge of nerve force through the pneumogastric nerves which throws the bronchial muscles into contraction. But whatever the intimate nature of this contraction may be it is evidence of nerve degradation of this contraction or nerve weakness, and strychnine by elevating the tone of these nerves increases the controlling power of the same.

A stimulant dose of strychnine will depend on the age of the patient and length of time during which the drug has been given, although asthmatics as a rule will bear larger doses of strychnine than most other patients. Begin, as a rule, with 1-30 of a grain subcutaneously once a day, and gradually increase to 1-20 or to 1-10 of a grain or more, if necessary to impress the system with its full stimulant effects. Do not waste your time with small doses. To these amounts of strychnine small doses of from 1-400 to 1-600 of a grain of atropine may be added. It is best to administer these drugs in the evening, because asthma is nocturnal in its attacks and your patient should be protected at night so he can sleep. Additionally to its hypodermic use this drug may be given in the following combination :

℞. Phenacetini, gr. lxiv.
Quiniæ sulph., gr. xxxii.
Ammon. muriat., ℥iss.
Pulv. capsici, gr. iv.
Strychniæ sulph., gr. 1⅛.
M. Ft. Capsulae No. xxxii.
Sig. One capsule four times daily.

Or in the following:

℞. .Strychniæ sulph., gr. 1⅛.
Syr. acid hydriodici,
Syr. hypophosph., aa. fl. ℥ij.
M. Sig. One teaspoonful four times daily.

In fact light cases of asthma require no hypodermic injection and do well enough when the above named preparations are given. In severe cases it is of course advisable to add morphine or nitroglycerine to the strychnine and atropine treatment, especially at the beginning. This treatment will break up the paroxysm, but even after they are broken many old asthmatics still remain in the most abject misery. They may be compelled to sit up day and night panting for breath, and still labor under the impression that they are suffering from asthma. This is a mistake, it is not asthma, but the natural state of exhaustion which follows asthma. The respiratory movements, as well as the whole nervous system, are almost completely paralyzed. It is the disorder and chaos following the flood. The dyspnœa is not paroxysmal as before, but is felt now on the slightest exertion. This stage of the disease is most important from a therapeutic standpoint. Nitroglycerine, lobelia and other narcotics are of no use. Rest is most essential now. They must do absolutely nothing. Lie down, if they can, or sit still. They should even be fed. I have known patients who were breathing comfortably to bring on a most severe exhaustion-dyspnœa by merely undertaking to write a letter. During the rest treatment give food of the most nourishing character, such as freshly expressed beef juice, a cupful a day, beef powder, beef, mutton, milk, oysters, clams, etc. In this stage strychnine is also of the greatest value. Massaging is also to be used in desperate cases. Electricity is also of great service. So are rarefied air and calisthenic exercises obtained in the pneumatic cabinet treatment. To procure sleep at night, morphine may be added to the hypodermic injections of strychnine. Success in treating asthma depends as much on the proper management of the individual as it does on the administration of drugs in the proper form.

